# Depressive symptoms, insomnia and dyspnea in COVID-19 survivors: a tunisian study

**DOI:** 10.1192/j.eurpsy.2024.1072

**Published:** 2024-08-27

**Authors:** B. Nadia, T. Mariem, B. A. Houda, C. Ghada, E. Sahar, M. Sameh, K. Sami, H. Najla, A. Jihen

**Affiliations:** ^1^Psychiatry B; ^2^Preventive medicine and hospital hygiene; ^3^Pneumology, Hedi Chaker university Hospital, Sfax, Tunisia

## Abstract

**Introduction:**

The coronavirus infection emerging in 2019 caused a plethora of physical and mental health problems around the world. Recent studies showed a persistent psychological distress even after few months of the infection.

**Objectives:**

To determine the prevalence of depressive symptoms, insomnia and dyspnea among covid-19 survivors.

**Methods:**

We conducted a prospective cohort study including 121 Tunisian COVID-19 inpatients who had been discharged alive from hospital. Each enrolled patient was asked about the period before the hospital stay, and the 6-9 month-period after hospital discharge. Patient Health Questionnaire-9 (PHQ-9) was used to assess depressive symptoms. We assessed *insomnia via the insomnia severity index (ISI) and dyspnea through the mMRC (*modified British Medical Research Council*).*

**Results:**

The median age of participants was 59 years. The prevalence of depressive symptoms and insomnia increased significantly after the pandemic (5.7% vs 57.9%, p=0.038, r=0.189; and 4.9% vs 26.4%; p<0.0001, r=0.349 respectively). Younger patients presented more depressive symptoms (p<0.0001). females were more likely to suffer from depressive symptoms (p<0.0001). Dyspnea was more prevalent among survivors with depressive symptoms (p=0.001). Patients with depressive symptoms exhibited more insomnia (p<0.0001).

**Conclusions:**

The pandemic of covid19 emerged a wide range of physical and mental health problems with complex physiopathology. The early detection of these disorders improves the quality of life of these patients.

**Disclosure of Interest:**

None Declared

